# Differential Remodelling of Endometrial Extracellular Matrix in the Non-Pregnant Uterus of *Lagostomus maximus* as a Potential Mechanism Underlying Embryonic Death

**DOI:** 10.3390/ani15040542

**Published:** 2025-02-13

**Authors:** Francisco Acuña, Gisela Soledad Gualdoni, Francisco Rivollier, Camila Barril, Enrique Leo Portiansky, Claudio Gustavo Barbeito, Elisa Cebral

**Affiliations:** 1 Laboratorio de Histología y Embriología Descriptiva, Experimental y Comparada, Facultad de Ciencias Veterinarias, Universidad Nacional de La Plata, La Plata CP1900, Buenos Aires, Argentina; franrivollier@gmail.com; 2 Consejo Nacional de Investigaciones Científicas y Técnicas (CONICET), La Plata CP1900, Buenos Aires, Argentina; elporti@fcv.unlp.edu.ar; 3 Laboratorio de Reproducción y Fisiología Materno-Embrionaria, Instituto de Biodiversidad y Biología Experimental y Aplicada (IBBEA), Consejo Nacional de Investigaciones Científicas y Técnicas (CONICET), Departamento de Biodiversidad y Biología Experimental (DBBE), Facultad de Ciencias Exactas y Naturales, Universidad de Buenos Aires, Ciudad Autónoma de Buenos Aires CP1428, Buenos Aires, Argentina; giselagualdoni@gmail.com (G.S.G.); camilabarril1995@gmail.com (C.B.); ecebral@hotmail.com (E.C.); 4 Laboratorio de Análisis de Imágenes, Facultad de Ciencias Veterinarias, Universidad Nacional de La Plata, La Plata CP1900, Buenos Aires, Argentina

**Keywords:** uterus, connective tissue, embryo loss

## Abstract

During development, the remodelling of fibrillar components of the uterine extracellular matrix (ECM), mediated by the matrix metalloproteinases (MMPs) and their tissue inhibitors (TIMPs), plays an essential role in embryonic survival. Previously, we observed that in the plains viscacha (*Lagostomus maximus*), only caudal implantation sites (IS) contain viable embryos, whereas embryos at cranial and middle IS die and are reabsorbed. The objective of this study was to analyse the distribution and expression of key components of the endometrial ECM, including fibrillar collagens, MMPs 2 and 9, and TIMPs 1 and 2, in three uterine segments (US) of the non-pregnant adult viscachas. In sections from three US, we observed a significant craniocaudal increase in collagen fibres (Van Gieson and Picrosirius red staining) and elastic fibres (Verhoeff-Van Gieson trichrome staining), along with the immunolabelling levels of MMP-2, MMP-9, TIMP-1, and TIMP-2 (immunohistochemistry). Zymography revealed similar gelatinolytic activity of MMP-2 in the three US but higher than the MMP-9 activity. However, MMP-9 activity in the caudal segment was significantly higher than that in the cranial and middle ones. These findings suggest that uterine ECM variations along the craniocaudal axis may contribute to uterine remodelling processes that regulate embryonic survival during gestation.

## Simple Summary

Embryonic death is an inherent process in the reproductive biology of the plains viscacha (*Lagostomus maximus*). During this process of embryonic death, embryos implanted in the caudal uterine segments survive, whereas those in the cranial and middle segments do not. Endometrial remodelling plays a key role in embryonic development. In this study, various extracellular matrix molecules (metalloproteinases, their inhibitors, and fibrillar components) were analysed in three uterine segments (cranial, middle, and caudal) of adult viscachas using histology, immunohistochemistry, and zymography. The results revealed greater endometrial remodelling along the craniocaudal axis. This finding may help to explain the phenomenon of embryonic death in this species.

## 1. Introduction

The gestation period of *Lagostomus maximus* (plains viscacha) is reported to be five months [1]. Embryonic implantation, which commences at 26 days post-coitus (dpc) [2], initiates in the caudal uterine segments (in proximity to each cervix), progresses in the middle segments, and culminates in the cranial segments (near the oviducts). Each uterine horn contains a total of five to six implantation sites (IS). Embryos located in the cranial and middle IS have been observed to die between 35 and 70 dpc, while embryos situated in the caudal IS survive [2,3,4]. Furthermore, this mortality rate is observed to be consistent, occurring at a similar rate (80%) and in the same uterine locations [5,6,7].

Previous morphological and morphometric studies of the uterine horns in non-pregnant anoestrus viscacha have revealed that the thickness of the uterine wall and its layers, as well as the areas occupied by blood vessels and uterine glands, increase in a craniocaudal direction [8]. This morphological variation in non-pregnant uterine horns was linked to the death and the survival of embryos [6,7]. For example, we infer that differential vascularisation along the uterine horns could influence the invasive placentation of each IS [8,9,10,11]. On the other hand, the study of the variation in endometrial thickness (which increases toward the caudal end of the uterine horns) has not been addressed. This variation could result in differential extracellular matrix (ECM) remodelling among the IS, leading to invasive placentation being insufficient in cranial and middle IS but optimal in caudal IS. Their study, in combination with previous results, would allow for a comprehensive understanding of the massive and localised embryonic death in the viscacha.

The ECM maintains the morphological integrity of the uterus, facilitating implantation, regulating trophoblastic invasion, and mediating the establishment of the maternal-embryo/foetal interface [12]. The ECM exhibits substantial structural and functional diversity [13]. A variety of factors induce qualitative and quantitative changes in the ECM composition, thereby rendering it a dynamic tissue structure [14,15]. The ECM is composed of collagen, reticular and elastic fibres, multiadhesive proteins, glycosaminoglycans, and proteoglycans [15,16]. Collagens, forming collagen and reticular fibres, identified by Picrosirius red staining, are the most abundant macromolecules in extracellular matrices [17].

On the other hand, the remodelling of the ECM involves the participation of several metalloproteinases (MMPs) and their tissue inhibitors (TIMPs) [18]. MMPs are zinc-dependent endopeptidases, and their proteolytic activities are subject to precise regulation at multiple levels, including the production and secretion of proenzymes, the activation of proenzymes, and the inhibition of MMPs by their own inhibitors. MMP-2 is responsible for the cleavage of several different proteins, including collagens (types I, II, III and IV), laminin, fibronectin, and elastin. MMP-9 is capable of degrading denatured collagens, as well as native types IV, V, XI, and XIV [16,19,20]. TIMPs are the primary endogenous inhibitors of MMPs, which exert their inhibitory effect by forming a 1:1 complex with the target enzyme. Four homologous TIMPs (TIMPs-1 to 4) have been identified [21]. TIMP-1 has been demonstrated to inhibit MMP-9, while TIMP-2 has been shown to inhibit MMP-2. MMPs are expressed in epithelial, stromal and glandular, as well as in immune and vascular cells of the uterus during normal oestrous and menstrual cycles, as well as during early pregnancy and decidualisation [22,23,24].

Considering the aforementioned context, we put forth the hypothesis that the uterus of the non-pregnant viscacha exhibits histomorphological variations related to differential topographical patterns of ECM components in the craniocaudal direction; these variations are a predetermined spatial regulatory mechanism for embryonic survival. The objective of this study was to undertake a histological and semiquantitative analysis of the content of collagen, elastic, and reticular fibres, evaluate the localisation, distribution, and immunoexpression of MMP-2, MMP-9, and TIMPs (1, 2), and determine the gelatinase activity of both MMPs in the cranial, middle, and caudal uterine segments of non-pregnant viscachas.

The viscacha model provides a valuable alternative for analysing morphological and molecular changes associated with embryonic death [7], with potential implications for studying both animal and human diseases, as well as addressing questions related to animal production.

## 2. Materials and Methods

### 2.1. Animal Capture

Five adult non-pregnant females of the species *Lagostomus maximus* were used, captured at the Wildlife Breeding Station (ECAS), Berazategui, Buenos Aires Province, between December and February. The simultaneous capture of all females during the mentioned period ensures the anestrus state. In this way, the reproductive organs were under the same influence of sex hormones. The histological characteristics of the uterine cervix and ovary were used to confirm the anestrus period [5,25]. The animal handling protocol (capture, anaesthesia, analgesia, and euthanasia) was based on previous studies [8] and approved by the Institutional Committee for the Care and Use of Laboratory Animals at the School of Veterinary Sciences, National University of La Plata (code 122-4-22P).

### 2.2. Sampling and Histological Processing

**Figure 1:**
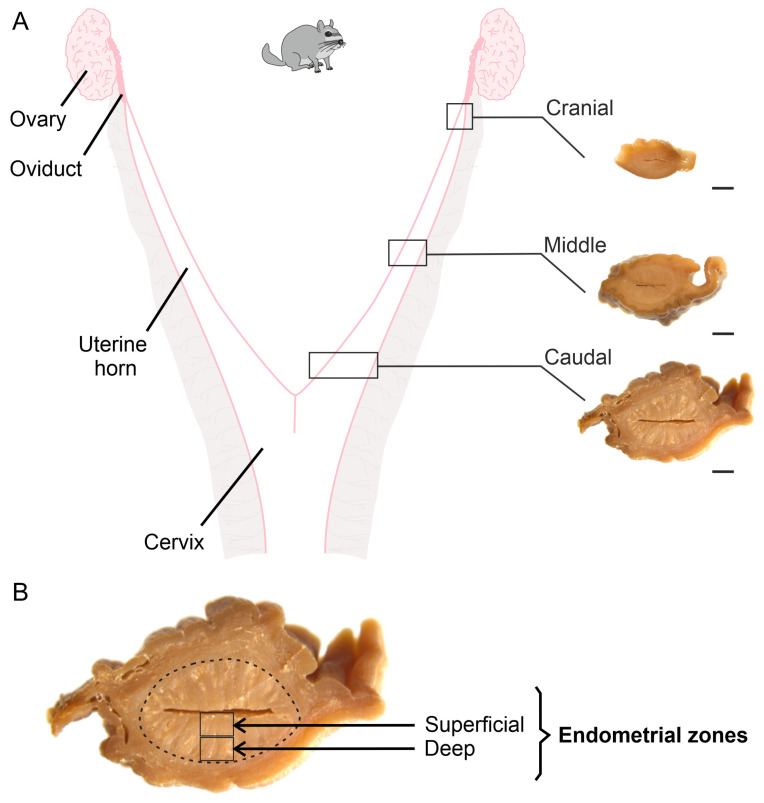
Schematic representation of the non-pregnant uterine horns of *L. maximus*. (**A**) On the right, macroscopic view of the cranial, middle, and caudal uterine segments. Scale bar: 1 cm. (**B**) Cross-sectional view of one of the uterine segments. The endometrium is circumscribed by a dashed line, with the superficial and deep endometrial zones identified.

Following euthanasia, the right and left uterine horns of each female were dissected and transversely sectioned along their craniocaudal longitudinal axis to obtain three 1-cm-long segments: the cranial segment (immediately adjacent to the utero-tubal junction), the middle segment (the central portion of the uterine horn, equidistant from the cranial and caudal ends), and the caudal segment (immediately adjacent to the utero-cervical junction) (Figure 1A) [8]. The three segments from the right uterine horn were processed using conventional histological techniques. Subsequently, the samples were then serially sectioned at a thickness of 5 µm [26]. The sections from each uterine segment were stained with conventional and special histological stains or prepared for immunohistochemical analysis. The three uterine segments from the left uterine horn of all females were weighed and then stored immediately at −80 °C for subsequent zymographic studies.

### 2.3. Histological Staining and Image Analysis

Sections from the three uterine segments were deparaffinised in xylene, rehydrated using a graded ethanol series in decreasing concentrations, and rinsed in distilled water. Haematoxylin-eosin staining was used for histological description and corroborates previous observations [8]. Van Gieson’s trichrome stain [27] was applied for collagen fibre analysis. The Picrosirius red staining was used to identify fibres composed of type I and III collagen [28], and the Verhoeff-Van Gieson staining was used to reveal elastic fibres [29,30] (Table 1). Subsequently, sections were dehydrated through an ascending ethanol series, cleared in xylene, and mounted with Canada balsam [8].

From the stained sections for each fibrillar ECM element in the three uterine segments, five images were captured at 40x magnification from each of the two endometrial zones (superficial and deep) (Figure 1B) using a digital camera (Leica ICC50W, Weztlar, Germany) attached to a bright-field microscope (Leica DM500, Weztlar, Germany). To quantify the content of collagen fibres (types I and III), as well as elastic fibres in the lamina propria of both endometrial zones, the software ImagePro Plus (v6.3, Media Cybernetics, Rockville, MD, USA) was employed. The region of interest (ROI) was defined, and its area (5213 µm^2^) was quantified. The area occupied (µm^2^) by the stained element within the ROI was then quantified, and the content of the element of interest was calculated using the following formula:

Staining Area% = (Stained Area/ROI Area) × 100

where Stained Area = area occupied by the stained fibrillar element.

The percentage of staining in the ROI for each zone of endometrial connective tissue and for the three uterine segments was calculated as the mean and standard deviation.

To analyse collagen types I and III in the connective tissue of the endometrium of uterine segments, Picrosirius red staining and polarised light microscopy were used to identify the yellow-red strong birefringence of collagen type I and the weak birefringence of greenish collagen type III.

Five images were captured from the lamina propria of the two endometrial zones at 40x magnification using a digital camera (Olympus DP73, Shinjuku, Japan) attached to a transmitted light microscope (Olympus BX53, Shinjuku, Japan) equipped with a polariser (U-POT) and an analyser (U-ANT). Subsequently, the content of each collagen type and the total collagen (types I + III) was quantified in relation to the total collagen (%), with respect to the ROI area, as well as the ratio of collagen I:III. The results were expressed as the mean percentage and standard deviation for the deep connective tissue zone in the three uterine segments.

### 2.4. Immunohistochemistry and Image Analysis

The immunohistochemistry protocol, previously employed in the species [7], was used in a standardised manner. Briefly, the sections were deparaffinised and rehydrated, and the endogenous peroxidase was then blocked using H_2_O_2_/methanol at room temperature. After washes with phosphate-buffered saline (PBS), nonspecific binding was blocked using 1% bovine serum albumin at room temperature. The sections were then incubated separately with each primary antibody (Table 2) overnight at 4°C. Following PBS washes, the sections were incubated with secondary antibodies (Table 2). Negative controls were used. For this, sections from the three uterine segments were incubated with immunoglobulin G (ready to use; Vector Laboratories BA1000, Burlingame, CA, USA) instead of each primary antibody. Finally, all sections were revealed using diaminobenzidine (DakoCytomation, Carpinteria, CA, USA), counterstained with Harris haematoxylin, and mounted with Canada balsam.

For the semiquantitative analysis of MMPs and their inhibitors immunolabelling, five image fields from bright-field microscopy at 40x magnification from both the superficial and deep endometrial zones for each uterine segment and for each antibody used were obtained. The ImagePro Plus software (v6.3, Media Cybernetics) was used to define a region of interest (ROI) and ascertain an area (µm^2^) for the epithelium (luminal and glandular) (2658 µm^2^) and lamina propria (5213 µm^2^). The area (µm^2^) occupied by the immunolabelled antigen within each area and for each tissue was then calculated, and the following formula was used:

Immunolabelled Area (%) = (Stained Area for the Antigen/ROI Area) × 100

The mean and standard deviation were subsequently calculated for the percentage values obtained from the five images per tissue of each animal (five females).

### 2.5. Gelatin Zymography

The zymography technique was used to assess the gelatinolytic activity of metalloproteinases MMP-2 and MMP-9. Uterine cranial, middle, and caudal segments (mean weight: 315.3 + 45.4 mg) from each female were homogenised in ice-cold lysis buffer [50 mM Tris-HCl (pH 7.4), 5 mM CaCl_2_, 1 µM ZnCl_2_, 1% Triton X-100], and incubated for 2 h. After centrifugation, the protein content of the supernatant was determined (Bradford assay). Tissue supernatants were mixed with the sample buffer (10 mM Tris-HCl, 0.06% bromophenol blue, 10% glycerol, 2% SDS) to be loaded in 10% SDS-PAGE-gel electrophoresis containing 1 mg⁄ml gelatin. To ascertain the presence of gelatinolytic MMP activity, a duplicate gel with each sample of each tissue supernatant was incubated in the presence of EDTA after electrophoresis. This was carried out for each sample of tissue supernatant, and the corresponding MMP bands disappeared, confirming the activity. After electrophoresis, the gels were washed in Tris-2.5% Triton X-100 and then in phosphate-buffered saline (PBS). The gels were incubated in the digestion buffer [50 mM Tris–HCl (pH 7.5), 0.15 M NaCl and 0.005 M CaCl2] at 37 °C for 24 h, and then stained (2.5% Coomassie brilliant blue R-250) for 45 min and destained (methanol: acetic acid: water (3:1:6). The gelatinase bands of MMP-2 and MMP-9 proteolytic activity in each uterine segment were identified by their molecular weight in accordance with the prestained MW protein standards (BioRad, Hercules, CA, USA). After photographing (Fujifilm LAS-1000 bio-imaging analyser, Fujifilm, Tokyo, Japan), the metalloproteinase gelatinolytic activities of each sample were evaluated as the densitometry of digestion band intensity by Image Scn software (Fujifilm LAS-100; Fujifilm, Tokyo, Japan). The data were expressed as mean arbitrary units (AUs)/µg protein ± standard deviation (SD) [24].

### 2.6. Statistical Analysis

The mean of the percent contents of the endometrial fibrillar elements, MMP and TIMP, and arbitrary units (AUs) of MMP activity levels from zymography of all females, were analysed by one-way analysis of variance (ANOVA), using Tukey’s multiple post hoc test, when the data satisfied assumptions of normality and variance homogeneity required for ANOVA. The statistical analyses were conducted using the Infostat software 2020I (https://www.infostat.com.ar/, accessed on 5 September 2024. Differences were considered significant when *p* < 0.05.

## 3. Results

### 3.1. Histology of Uterine Segments and Fibrillar Component Content in the Connective Tissue of Superficial and Deep Endometrial Zones

**Figure 2:**
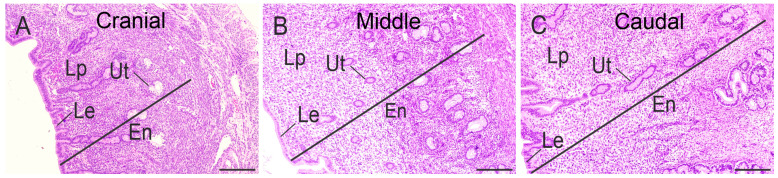
Histology of the endometrium of the cranial (**A**), middle (**B**), and caudal (**C**) uterine segments of non-pregnant *L. maximus*. Haematoxylin-eosin. Scale bar: 500 µm. Abbreviations: En, endometrium; Le, luminal epithelium; Lp, lamina propria; Ut, uterine glands.

As was observed in our previous studies in the non-pregnant vizcacha uterus [8], a series of morphological differences and an increase in the thickness of both uterine horns were observed from cranial to caudal. Histologically, the three uterine tunics (endometrium, myometrium, and perimetrium) were present in all three cranial-caudal segments. In comparison, the thickness of the endometrium was observed to increase from the cranial to the caudal uterine segment (Figure 2A–C).

**Figure 3:**
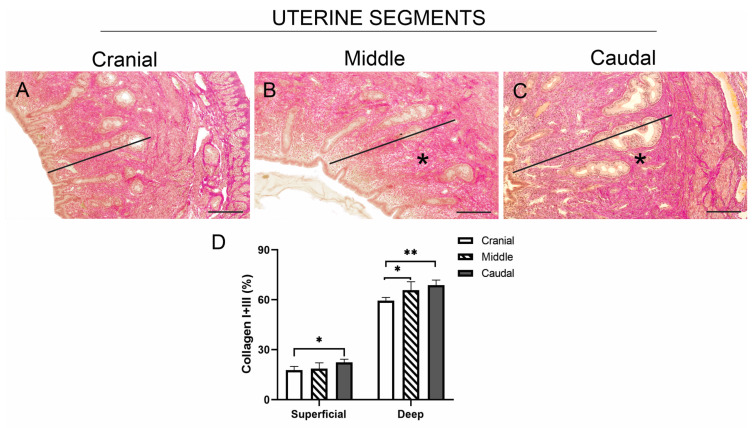
Representative images of sections from the three uterine segments of non-pregnant *L. maximus*, stained with Van Gieson to identify collagen fibres in the connective tissue of the endometrium (solid line). (**A**) Image showing collagen fibres homogeneously distributed throughout the thickness of the endometrium in the cranial uterine segment. (**B**,**C**) Images showing collagen fibres predominantly distributed in the deep region (asterisk) of the endometrium in the middle (**B**) and caudal (**C**) segments. (**D**) Percentage (mean and standard deviation (SD) of collagen fibre area stained with Picrosirius in the superficial and deep endometrial zones of the three uterine segments. Significance: * *p* < 0.05, ** *p* < 0.01. Scale bar: 200 µm.

The distribution and content of the main fibrillar components in the connective tissue of the superficial and deep endometrial zones were analysed across the three uterine segments. Using Van Gieson trichrome staining, collagen fibres (red-pink) were distributed throughout the entire thickness of the endometrial connective tissue in the cranial uterine segment (Figure 3A), but were more concentrated in the depth of the endometrium in the middle and caudal segments (Figure 3B,C). This same pattern was observed using Picrosirius red staining observed with bright-field microscopy (Figure S1).

The content of collagen fibres in the uterine sections stained with Van Gieson was determined (Figure 3D). When comparing the collagen fibre content in the superficial versus deep zones of the three uterine segments, it was observed that the deep endometrial zone contained more collagen fibres than the superficial zone. The superficial zone of the caudal segment had a significantly higher collagen fibre content than the same zone in the cranial segment (*p* < 0.05). The deep zone of the endometrium in both the middle and caudal segments also had a significantly higher collagen fibre content compared to the deep endometrium of the cranial segment (*p* < 0.05 and *p* < 0.01).

**Figure 4:**
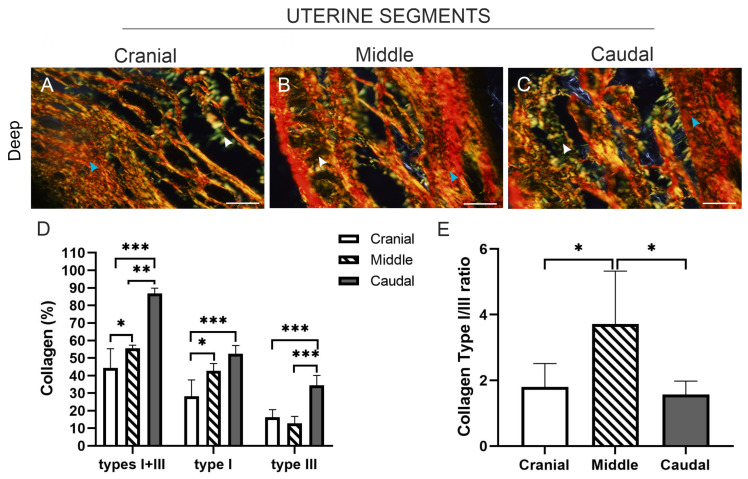
Representative images of histological sections from the three uterine segments of non-pregnant *L. maximus*, stained with Picrosirius red and observed under polarised light microscopy to identify collagen fibres of types I and III in the connective tissue of the endometrium. (**A**–**C**) Images of collagen fibres in the deep connective tissue of the endometrium from the three uterine segments. Type I collagen (red-yellow, blue arrowhead) and type III collagen (green, white arrowhead). (**D**) Percentage (mean and SD) of staining area for type I and III collagen in the deep connective tissue of the endometrium across the three uterine segments. (**E**) Type I/III collagen ratio in the connective tissue of the deep endometrial zone in the three uterine segments. Significance: * *p* < 0.05; ** *p* < 0.01; *** *p* < 0.001. Scale bar: 200 µm.

We also analysed the organisation and relative content of type I and type III collagen fibres in sections of the three uterine segments stained with Picrosirius red and observed under polarised light microscopy. In the deep endometrium of the cranial segment (Figure 4A), both type I (red/yellow) and type III (green) collagen fibres displayed an organised, uniform, or regular fibrillar structure. In the middle and caudal segments, the distribution of these fibres in the ECM showed a more disorganised pattern, especially in the caudal segment, where there was greater fibrillar cross-linking (Figure 4B,C).

A semiquantitative analysis was performed to determine the content of type I and type III collagen fibres in the connective tissue of the deep endometrium (Figure 4D,E) in sections of the three uterine segments stained with Picrosirius red and observed under polarised light microscopy. The total percentage of collagen types I and III exhibited an increase from the cranial to the caudal segment, being significantly higher in the caudal segment relative to the other two segments (*p* < 0.01, *p* < 0.001). The percentage of type I collagen followed the same pattern as the total collagen content. The caudal and middle segments had significantly higher percentages of type I collagen compared to the cranial segment (*p* < 0.001, *p* < 0.05). The content of type III collagen was significantly higher in the caudal segment in comparison to the middle (*p* < 0.001) and cranial (*p* < 0.001) segments. No differences were observed in the type III collagen content between the middle and cranial segments (Figure 4D). Moreover, the ratio of type I to type III collagen exhibited a significant increase in the middle segment when compared to both the cranial and caudal segments (*p* < 0.05) (Figure 4E).

**Figure 5:**
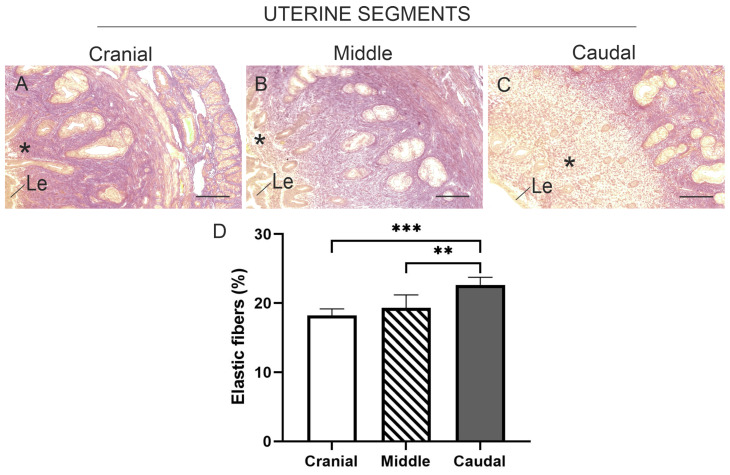
Representative images of sections from the three uterine segments of non-pregnant *L. maximus*, stained with Verhoff-Van Gieson to visualise elastic fibres in the connective tissue of the endometrium. (**A**–**C**) Localisation (asterisk) of elastic fibres in the superficial endometrium in the cranial (**A**), middle (**B**), and caudal (**C**) uterine segments. (**D**) Percentage (mean and SD) of staining area for elastic fibres in the superficial endometrium across the three analysed segments. Significance: ** *p* < 0.01; *** *p* < 0.001. Scale bar: 500 µm. Abbreviations: Le, luminal epithelium.

In all three uterine segments, elastic fibres were primarily localised and distributed in the connective tissue of the superficial endometrium, while the deep zone showed a faint staining (Figure 5A–C). In the superficial endometrium, the content of elastic fibres did not differ between the cranial and middle segments, but it significantly increased in the tissue of the caudal segment, with significant differences observed between the caudal segment and both the middle and cranial segments (*p* < 0.01 and *p* < 0.001, respectively) (Figure 5D).

### 3.2. Immunolocalisation and Content of MMP-2 and MMP-9 in the Endometrial of the Uterine Segments

**Figure 6:**
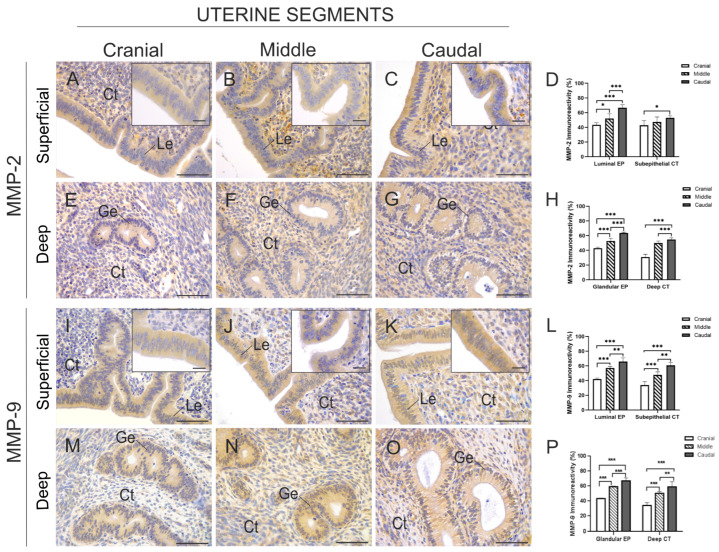
Immunoexpression and semi-quantification of MMP-2 and MMP-9 in the superficial and deep endometrial zones of the cranial, middle, and caudal uterine segments of *L. maximus*. (**A**–**C**) Immunostaining of MMP-2 in the luminal epithelial tissue and connective tissue of the superficial endometrium from the cranial (**A**), middle (**B**), and caudal (**C**) uterine segments. Inserts show high magnification of the epithelium. (**D**) Quantification of the percentage of immunostained area of MMP-2 in the luminal epithelium and connective tissue of the superficial endometrium across the three uterine segments. (**E**–**G**) Immunostaining MMP-2 in the glandular epithelial tissue and connective tissue of the deep endometrium in the cranial (**E**), middle (**F**), and caudal (**G**) uterine segments. Inserts show high magnification of the epithelium. (**H**) Percentage of immunostained area of MMP-2 in the glandular epithelium and connective tissue of the deep endometrium across the three uterine segments. (**I**–**K**) Immunostaining of MMP-9 in the luminal epithelial tissue and connective tissue of the superficial endometrium in the cranial (**I**), middle (**J**), and caudal (**K**) uterine segments. (**L**) Percentage of immunostained area of MMP-9 in the aforementioned tissues in the superficial endometrium of the three analysed uterine segments. (**M**–**O**) Immunostaining of MMP-9 in the glandular epithelial tissue and connective tissue of the deep endometrium in the cranial (**M**), middle (**N**), and caudal (**O**) uterine segments. (**P**) Percentage of immunostained area of MMP-9 in the aforementioned tissues in the deep endometrium of the three analysed segments. Significance: * *p* < 0.05; ** *p* < 0.01; *** *p* < 0.001. Scale bar: 200 µm (**A**–**C**,**E**–**G**,**I**–**K**,**M**–**O**), 20 µm (Inserts). Abbreviations: Ct, connective tissue; Ge, glandular epithelium; Le, luminal epithelium.

Figure 6 shows the immunostaining of MMP-2 and MMP-9 in the endometrial histological structures of the three uterine segments. The cytoplasmic staining of MMP-2 was localised in the luminal and glandular epithelia, while in the connective tissue, it was present in the cytoplasm of fibroblasts and in the extracellular matrix (Figure 6A–G).

In the superficial endometrium, the immunostained area of MMP-2 of the luminal epithelium and subepithelial connective tissue increased from the cranial to the caudal uterine segment, with a significantly higher MMP-2 content in the caudal segments than in the cranial and middle ones (*p* < 0.001) (Figure 6D). The percentage of immunostained area of MMP-2 in the glandular epithelium was quantified in the deep endometrial tissue, demonstrating a significant increase from cranial to caudal. The caudal segment exhibited a higher level of immunostained area compared to the cranial and middle segments (*p* < 0.001). In the deep connective tissue, a significantly higher percentage of immunostained MMP-2 was observed in the caudal segment compared to the cranial and middle segments (*p* < 0.001) (Figure 6H).

The immunoexpression of MMP-9 was also observed in the same endometrial structures as MMP-2, both in the superficial endometrium (Figure 6I–K) and in the deep endometrium (Figure 6M–O). The percentage of immunostained area of MMP-9 in the luminal epithelium and connective tissue of the superficial endometrium of the caudal segment was significantly higher than in these tissues in the cranial and middle segments (*p* < 0.001) (Figure 6L). In the glandular epithelium and connective tissue of the deep endometrium, the same pattern of increasing MMP-9 content in the cranial to caudal direction was observed (*p* < 0.001) (Figure 6P). Negative controls for MMP-2 and MMP-9 are shown in Figure S2.

### 3.3. Immunolocalisation and Content of Metalloproteinase Inhibitors (TIMP) in the Endometrium of the Uterine Segments

**Figure 7:**
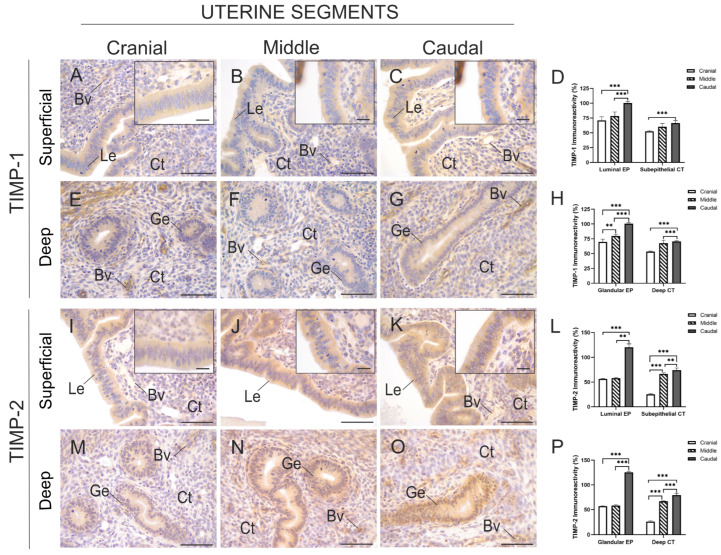
Immunostaining and semi-quantification of TIMP-1 and TIMP-2 in the superficial and deep endometrial zones of the cranial, middle, and caudal uterine segments of *L. maximus*. (**A**–**C**) Immunostaining of TIMP-1 in the luminal epithelial tissue, connective tissue, and blood vessels in the superficial endometrium of the cranial (**A**), middle (**B**), and caudal (**C**) uterine segments. Inserts show high magnification of the epithelium. (**D**) Percentage of immunostained area of TIMP-1 in the luminal epithelial and connective tissues of the superficial endometrium across the three analysed segments. (**E**–**G**) Immunostaining of TIMP-1 in the glandular epithelial tissue, connective tissue, and blood vessels in the deep endometrium of the cranial (**E**), middle (**F**), and caudal (**G**) uterine segments. (**H**) Percentage of immunostained area of TIMP-1 in the aforementioned tissues in the deep endometrium across the three analysed segments. (**I**–**K**) Immunostaining of TIMP-2 in the luminal epithelial tissue, connective tissue, and blood vessels in the superficial endometrium of the cranial (**I**), middle (**J**), and caudal (**K**) uterine segments. Inserts show high magnification of the epithelium (**L)** Percentage of immunostained area of TIMP-2 in the aforementioned tissues in the superficial endometrium across the three analysed segments. (**M**–**O**) Immunostaining of TIMP-2 in the glandular epithelial tissue, connective tissue, and blood vessels in the deep endometrium of the cranial (**M**), middle (**N**), and caudal (**O**) uterine segments. (**P**) Percentage of immunostained area of TIMP-2 in the aforementioned tissues in the deep endometrium across the three analysed segments. Significance: ** *p* < 0.01; *** *p* < 0.001. Scale bar: 200 µm (**A**–**C**,**E**–**G**,**I**–**K**,**M**–**O**), 20 µm (Inserts). Abbreviations: Bv, blood vessels; Ct, connective tissue; Ge, glandular epithelium; Le, luminal epithelium.

Figure 7 shows the immunolocalisation of TIMP-1 and TIMP-2 in the endometrium of the three uterine segments. The localisation of TIMP-1 in the superficial (Figure 7A–C) and deep (Figure 7E–G) endometrial zones of all three uterine segments was similar to that observed for both MMPs, although it was also found in the endothelium of blood vessels of varying calibres. In the superficial endometrium of the caudal segment, the percentage of TIMP-1 immunolabelled area in the luminal epithelium was significantly greater than in the middle and cranial segments (*p* < 0.001). In the subepithelial connective tissue, the content of TIMP-1 was significantly higher than in the cranial segment (*p* < 0.001) (Figure 7D). In the deep endometrium, significant differences were observed in the TIMP-1 content of the glandular epithelium between the three uterine segments. In the connective tissue of the deep endometrium, the TIMP-1 content was significantly higher in the caudal segment compared to the middle and cranial segments (*p* < 0.001) (Figure 7H).

The immunolocalisation of TIMP-2 was like that of TIMP-1. The quantification of the percentage of TIMP-2 immunolabelled area in the luminal epithelium of the superficial endometrium significantly increased only in the caudal segment when compared to the cranial and middle segments (*p* < 0.001 and *p* < 0.01, respectively). The TIMP-1 content was not significantly different between the cranial and middle segments. In the subepithelial connective tissue of the superficial endometrial zone, the percentage of immunolabelled area significantly increased across the segments, being higher in the caudal segment compared to the cranial and middle segments (Figure 7L). The same patterns of TIMP-2 content were observed in the glandular epithelium and connective tissue of the deep endometrium of the uterine segments (Figure 7P). Negative controls for both TIMPs are shown in Figure S3.

The localisation and content of TIMP-3 and TIMP-4 did not differ from what was observed for TIMP-2 in the same tissues of the three uterine segments. The results are shown in Figure S4 and the negative controls in Figure S5.

### 3.4. Relationships Between MMP and TIMP Content in the Endometrial Regions

The objective was to undertake a comparative analysis of the percentage of total immunolabelling of each metalloprotease in the superficial and deep endometrium, and to investigate the potential immunolabelled relationships and balances between MMPs/TIMPs.

**Figure 8:**
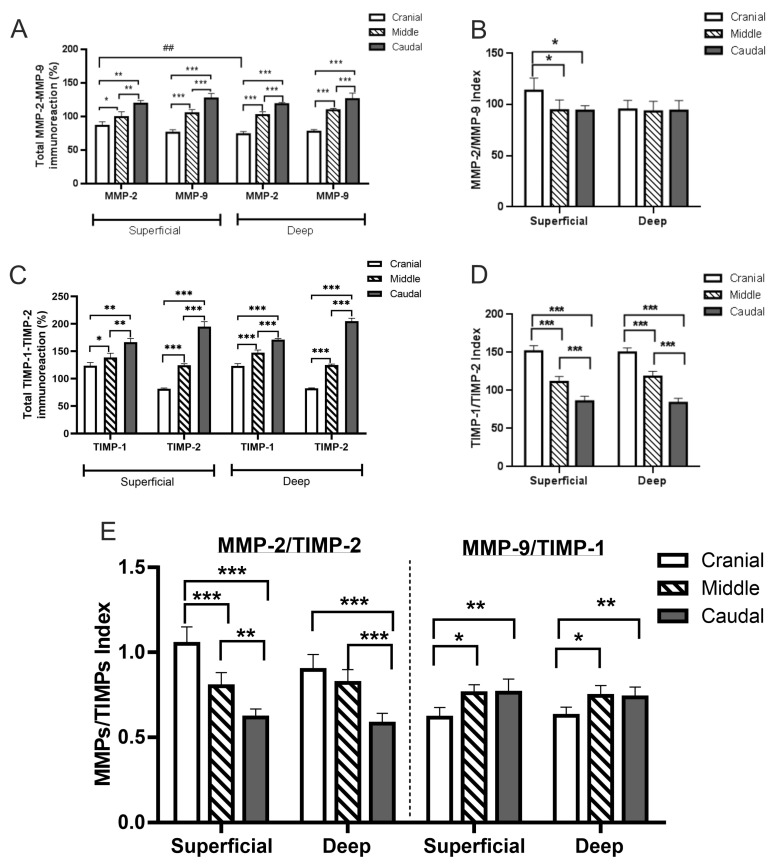
Total content of MMPs and their inhibitors and balances between MMPs and TIMPs in the endometrium of the cranial, middle, and caudal uterine segments of *L. maximus*. (**A**,**C**) Total content (mean percentage of immunostained area ± SD) of MMP-2, MMP-9, TIMP-1, and TIMP-2 in the superficial and deep endometrium of the cranial, middle, and caudal uterine segments. (**B**) Balance between MMPs expressed as the mean MMP-2/MMP-9 index (±SD). (**D**) Mean TIMP-1/TIMP-2 index (±SD). (**E**) Mean MMPs/TIMPs indices (±SD). Significance: * *p* < 0.05, ** *p* < 0.01, *** *p* < 0.001, ## *p* < 0.001.

The total percentage content of MMP-2 and MMP-9 increased significantly in both the superficial and deep endometrium from the cranial to caudal segments. The highest immunolabelling for both MMPs was observed in the superficial and deep endometrium of the caudal segment (Figure 8A). Then, the MMP-2/MMP-9 index showed an approximate 20% increase in the superficial endometrium of the cranial segment relative to the other tissues, where it exceeded one of the superficial endometria of the middle and caudal segments (Figure 8B). In contrast, the levels of the MMP-2/MMP-9 index in the deep endometrium of the uterine segments were similar to each other (Figure 8B).

The percentage of immunolabelling area of TIMP-1 and TIMP-2 exhibited a similar trend, demonstrating a notable increase across the cranial-middle, mid-caudal, and cranial-caudal uterine segments, both in the superficial and the deep endometrium (Figure 8C). A high percentage of immunolabelling area of both TIMP-1 and TIMP-2 was observed in the superficial and deep endometrium of the caudal segment. However, the percentage of TIMP-2 was lower in the superficial and deep endometrium of the cranial and middle segment when compared to the percentage of TIMP-1 in the same endometrial tissue zones of the aforementioned segments. The TIMP-1/TIMP-2 index was found to decrease from the cranial to the caudal segments, in both the superficial and the deep endometrium (Figure 8D).

The balance expression between each MMP and their inhibitors is vital for controlling tissue remodelling. The ratio of MMP-2 to TIMP-2 exhibited a similar trend with a decline from the cranial to the caudal segments observed in both the superficial and the deep endometrium (Figure 8E). The MMP-2/TIMP-2 index was observed to be significantly higher in the cranial segment of the superficial endometrium in comparison to the deep segment. The increment in the MMP-2/TIMP-2 index was approximately 15% and 23% in the cranial superficial endometrium in comparison to the other segments. The MMP-9/TIMP-1 indexes in both the superficial and deep endometrium of the three uterine segments were found to be similar to each other and lower than the MMP-2/TIMP-2 indexes (Figure 8E).

### 3.5. Gelatinase Activity of MMP-2 and MMP-9 in Endometrial Uterine Segments

**Figure 9:**
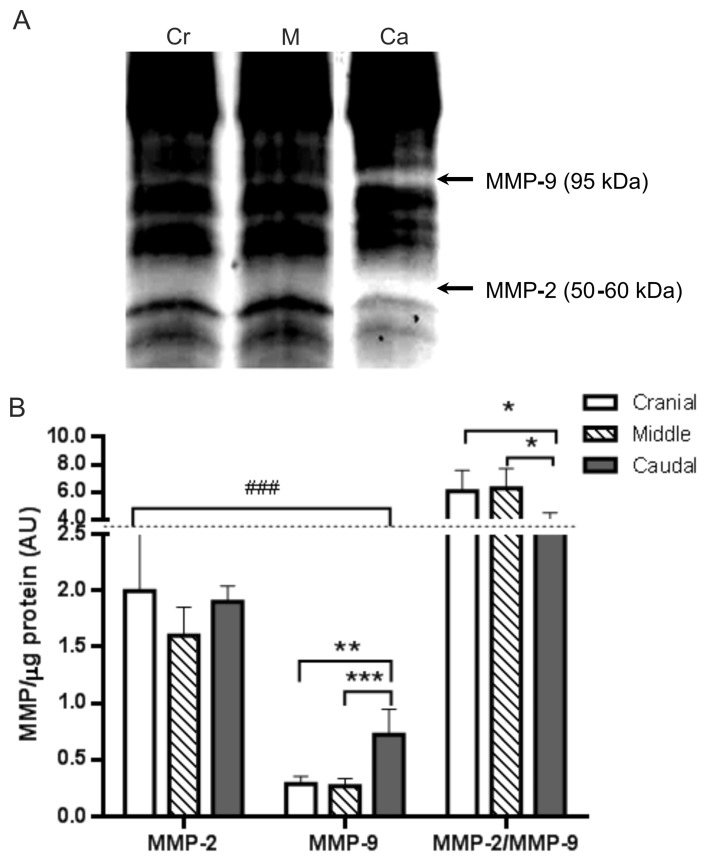
Gelatinolytic activity of MMP-2 and MMP-9 in cranial, middle, and caudal uterine segments of non-pregnant *L. maximus*. (**A**) Representative zymography of MMP-2 and MMP-9 in endometrial tissue samples from cranial (Cr), middle (M), and caudal (Ca) uterine segments, showing the active forms of MMP-9 in the 95 kDa molecular weight range and MMP-2 in the 50–60 kDa range. (**B**) Figure shows endometrial MMP-2 and MMP-9 activity levels, expressed as average arbitrary units (AUs) and standard deviation (SD) of active gelatinolytic MMP bands, as well as the ratio of MMP-2/MMP-9 activity in the cranial, middle, and caudal segments of the endometrium. Significance: * *p* < 0.05, ** *p* < 0.01, *** *p* < 0.001, ### *p* < 0.001.

Given the quantitative differences observed in the distribution and quantity of collagen and elastic fibres, both substrate for MMP-2 and MMP-9, and considering the changes in the labelling percentage of MMP and the inhibitors in the superficial and deep endometrium of uterine segments, we characterised and quantified the activity of MMP-2 and MMP-9 in the endometrium of three uterine segments by zymogram. As shown in Figure 9A, specific MMP bands corresponding to gelatinases MMP-9 (95 kDa) and MMP-2 (50–60 kDa) were observed in the cranial, middle, and caudal segments.

The semi-quantification of the respective bands of each MMP in the uterine segments revealed that the levels of MMP-2 enzymatic activity of three uterine segments were similar (Figure 9B). The levels of gelatinolytic activity of MMP-2 in the three uterine segments were found to be significantly higher than the corresponding levels of MMP-9 (*p* < 0.001). MMP-9 activity was significantly higher in the caudal segment in comparison to the cranial and middle segments (*p* < 0.001). Finally, the ratio of MMP-2/MMP-9 activities was similar between the cranial and middle segments but decreased significantly in the caudal segment when compared to the other two segments (*p* < 0.05) (Figure 9B).

## 4. Discussion

Gestational loss due to embryonic death in livestock species generates low reproductive performance [31] and, consequently, economic losses [32]. However, general mechanisms of embryonic death are not fully known. Compared to other models of embryonic death, the plains viscacha constitutes a very valuable alternative study tool for addressing the causes and mechanisms involved in early embryonic survival and mortality [10]. In this species, embryonic death, which is part of its reproductive biology, is characterised by being: 1. Spontaneous, since it is not caused by external aetiological agents [4]; 2. Sectorised, since the death of the embryos occurs in the cranial and middle implantation sites [3]; 3. Massive, since the embryonic death rate is 80% [6]; and 4. Embryonic death is morphologically similar to that observed in conventional models [5,6,7].

Optimal development of the embryo requires a balance between intrinsic factors, which are specific to the embryo, and extrinsic factors, which correspond to the maternal environment [33], which is determined by morphological, histological, and molecular characteristics of the uterine endometrium [34]. During early pregnancy, even before implantation, the uterine endometrium undergoes major changes to maintain the homeostasis and physiological conditions of the luminal and glandular epithelium and connective tissue [35]. In several groups of mammals, insectivores, bats, and South American camelids, greater vascularisation and expansion of the endometrial glands and the size of the uterine horns are found in specific segments (cranial, middle, or caudal) of one or both uterine horns with developing implantation sites [36,37,38,39]. In the viscacha, we have recently observed that five to six IS arranged in the caudal region of each uterine horn undergo viable development [5,34], while there is a greater vascularisation in the uterine caudal region of the uterus at 70 and 90 gestational days [6,34]. A similar pattern of uterine craniocaudal vascularisation has been found in non-pregnant adult females compared to pregnant viscacha uteri [8], and a correlatively increased thickness of the uterine tunics and glands in the craniocaudal direction of the non-pregnant viscacha uteri has recently been reported [8]. This differential vascularisation along the uterine horns may possibly influence hemotrophic nutrition and the placentation of each IS, favouring those located caudally [6].

Considering these results, and because some studies in other species have shown that unremodelled uterine structures and ECM cause abnormal embryo-placental development [40] leading to pregnancy loss due to embryonic death [41,42], here we hypothesised that the survival of implanted embryos along the uterine horns in viscacha, associated with morphological variations in uterine structures, may be related to a differential pattern of spatial distribution and expression of key molecular compounds of the uterine ECM along the craniocaudal direction of the non-pregnant uterus.

In addition to its function in the formation of cell and organ shape and volume, the ECM plays a pivotal role in regulating metabolic processes. It influences cellular proliferation, differentiation, and apoptosis, and serves as a reservoir for biologically active growth factors. In the ECM, the most abundant uterine collagen types I, III, and IV are subject to a constant remodelling process, whereby events of deposition, degradation, or other modifications occur simultaneously. To date, there are no reports on the arrangement and content of collagen and elastic fibres between the craniocaudal segments of non-pregnant uterine horns in the viscacha, nor in other species of multiparous mammals. This study demonstrates that the morphological variation in the glandular area and vascularisation of the non-gravid uterus of viscacha [6] is accompanied by a similar craniocaudal change in the fibrillar components of the ECM of the connective tissue of the deep and superficial endometrium. Initial observations revealed a higher quantity of collagen fibres in the deep endometrium than in the endometrial superficial tissue across all three uterine segments. However, the quantity of collagen fibres was significantly increased in the superficial and deep endometrium of the caudal segments in comparison to those tissues in the cranial one. These differences in the increasing abundance of collagen fibres in the endometrial ECM, starting from the middle uterine segment, may be attributed to a greater synthesis of collagens in the caudal direction. Therefore, in view of its potential predominantly structural role in the development of the glandular and vascular components of the endometrium, these collagen fibrillar components in the deep connective tissue follow the same craniocaudal distribution of the main endometrial structures observed in the non-pregnant and pregnant uterus of the viscacha [8].

Picrosirius red staining viewed by polarised microscopy was used to analyse the arrangement and relative content of collagen type I and III fibres in the three uterine segments. In the deep endometrium of the cranial segment, collagen types I and III showed a regular fibrillar structural aspect. However, in the middle and caudal segments, a pattern of an apparent greater disorganisation with a network aspect of these fibres was observed; this was more marked in the caudal segment, where greater fibrillar cross-linking was present. The disorganised appearance of type I and III collagen fibres in the caudal segment could be due: (a) a greater degree of denaturation or degradation of these collagen fibres; or (b) conversely, a greater network organisation of collagen I and III fibres. Regarding the latter interpretation, the amount of polarised light absorbed by the Picrosirius dye (which binds specifically along the collagen fibre) is strictly dependent on the orientation of the fibre bundles, meaning that a greater organisation of the collagen fibre implies a greater absorption of light by the Picrosirius dye. Therefore, it was proposed that Picrosirius red is particularly useful for revealing the molecular order, organisation, and/or heterogeneity of collagen fibre orientation. Many authors reported that the polarised colours of Picrosirius red staining depend only on fibre thickness and packing, but not on the composition of the specific collagen type within the collagen bundles [43,44,45]. However, this staining technique and morphometric image analysis was proposed as the most powerful method to quantify collagen network remodelling [46,47]. Consistent with the amount of collagen fibres in the three uterine segments observed by Van Gieson staining and Picrosirius red staining observed under polarised light, the total percentage of collagen type I + III increased from the cranial to the caudal uterine segment, with the significantly highest content in the caudal segment. In addition, while the caudal and middle segments had higher levels of collagen I than the cranial segment, collagen type III was highest only in the caudal segment. Considering that both a high relative abundance of collagen fibres and a correct fibrillar orientation and organisation correlate with a high absorption of polarised light by the dye, we think that the increased type III collagen (reticular), which promotes the formation of collagen fibres in the endometrial caudal segment, may give the typical fibrillar network appearance structure observed as a disorganised fibrillar-looking pattern in the deep endometrium of the caudal segment. If the collagen fibres had a low amount of type III collagen and were denatured, the structural arrangement of these fibrils could also be observed as a disorganised appearance, in which case the absorption of polarised light by the dye would be reduced and therefore a lower level of collagen type content would be detected in the caudal endometrium. However, this does not appear to be the case. We therefore believe that there is an active synthesis of type I collagen in the three uterine segments, with the highest levels in the caudal segment, but active synthesis of collagen III occurs only in the caudal segment, the collagen necessary for the formation of the network-type collagen fibres. In this respect, the collagen bundles are heterotypic fibres composed, in the non-pregnant uterus, of collagen types I and III, which are closely associated, with one predominating over the other. Thus, a loose packing of collagen fibres implies a higher content of collagen type I, while a more compact one has a greater amount of type III to form networks and develop a more compact ECM for structural functions. On the other hand, collagen type I has been associated with a reduction in cell adhesion and the promotion of cell proliferation [48]. Since we observed a higher collagen I/type III ratio in the middle segment, because of a greater synthesis of collagen I than of collagen III, perhaps this increased I/III ratio in the middle segment may play a particular role in the development of the ECM and connective structures to prepare the endometrium from the middle to the caudal segment. In the caudal segment, the rate of synthesis and/or degradation of each type of collagen may then reach a state of equilibrium. In this regard, functionally, collagen I could be an essential endometrial component necessary for collagen fibre conformation, and in the caudal segment, the increased collagen I with respect to the cranial one would be related to the assembly of the reticular structure of the network-type collagen fibres, which is necessary in the ECM as a supporting component for the development of glands and vessels in this endometrial zone. Therefore, we believe that due to the increased collagen I/III balance in the middle segment, the connective tissue may be less packed by reduced cell adhesion, a condition that would allow for a higher rate of cell proliferation of uterine components, such as the epithelia, ECM cells, and vascular endothelium. This could allow for an increasing development of endometrial structures from the middle segment towards the caudal segment, where there is the greatest abundance of glandular and vascular structures. Thus, these endometrial dynamic events and changes would possibly prepare and adapt the caudal endometrium for a potentially higher implantation rate and embryonic survival.

The same pattern of increase in the distribution and content of elastic fibres was observed in the craniocaudal direction of the endometrium. Based on the structural and physiological role of elastic fibres in the uterus [49], we think that their highest content in the caudal segment may participate in the uterine motility to transport gametes and embryos, in the latter case contributing to the spacing of embryos and their implantation.

During the non-pregnant cycle of the viscacha’s uterus, active ECM remodelling requires both highly coordinated degradation and denaturation of fibrillar collagens and rebuilding of the ECM by collagen resynthesis, in which MMPs play a key role [50]. In many adult tissues, the expression level of MMPs is very low, but it increases during active tissue remodelling. In humans, uterine MMPs, which show cyclical changes, play a relevant role in the remodelling of the endometrium during the menstrual cycle [22,51,52]. To date, there are no studies on the expression of MMP-2 and MMP-9 in all uterine segments in non-pregnant viscacha. Like the distribution and content of ECM fibrillar components along the endometrial segments of the non-pregnant uterus of viscacha, we propose a similar pattern of variation in the expression and activities of MMPs along the craniocaudal uterus.

In the luminal and glandular epithelium and in the ECM of the deep endometrial connective tissue of the caudal segment, we detected an increased immunoexpression of MMP-2 and MMP-9 compared to the same tissues in the middle and cranial segments. However, the balance of MMP-2/MMP-9 expression was increased in the superficial endometrium of the cranial segment compared to the other two segments. On the one hand, the increase in this ratio of MMPs in the superficial zone of the cranial segment may indicate a preponderant expression and activity of MMP-2 over MMP-9, and therefore in the probable degradation rate of its main substrates, the collagen types I and III and elastin, components that were observed to be reduced in the cranial uterus compared to the same zone of the caudal segment. Consistent with the increased endometrial gelatinolytic activity of MMP-2 compared to MMP-9 activity, we detected a higher ratio of MMP-2/MMP-9 gelatinolytic activity in the endometrium of the cranial segment compared to the caudal one. Furthermore, analysis of the MMP-2/TIMP-2 expression ratio indicates an increase in the cranial endometrium. On the other hand, the ratio of MMP-9/TIMP-1 expression tends to decrease in the cranial segment. Consequently, we believe that in the cranial endometrium the degree of degradation of the fibrillar components studied in this work is higher than in the caudal segment, and therefore the rate of remodelling of superficial and deep endometrial ECM is greater. In contrast, the higher content of collagen and elastic fibres, and probably a higher organisation/arrangement of collagen fibres, as mentioned above, in the connective tissue of the caudal segment of the endometrium suggest a lower rate of degradation of these fibres. This idea can be related to the lower MMP-2/MMP-9 activity ratio found in the caudal segment compared to the cranial and middle segments, suggesting a possible higher modulation of MMP-9 activity in the caudal segment. In addition, negative regulation of MMP-9 activity may be provided by the increased expression of its inhibitor TIMP-1, as evidenced by an increased MMP-9/TIMP-1 ratio in the caudal segment compared to the cranial segment. Inhibition results from the TIMP protein binding to the active site of MMPs in a 1:1 stoichiometric ratio, and therefore the balance or imbalance of MMPs with their inhibitor levels, is a potential predictor of ECM production and/or degradation.

In the luminal and glandular epithelium and in the ECM of the deep endometrial connective tissue of the caudal segment, we detected an increased immunoexpression of MMP-2 and MMP-9 compared to the same tissues in the middle and cranial segments. However, the balance of MMP-2/MMP-9 expression was increased in the superficial endometrium of the cranial segment compared to the other two segments. On the one hand, different studies performed in tissues and cell types from both diabetes and hyperglycaemia models show overexpression together with overactivity of MMPs [53,54,55], denoting correlations between their expression and activity. Therefore, the increase in this MMPs expression ratio in the superficial zone of the cranial segment may suggest a preponderant activity of MMP-2 over MMP-9. Thus, a probable degradation rate of collagen types I and III and elastin, the main substrates of MMP-2, may occur in the cranial uterus where they are observed to be reduced compared to the same zone of the caudal segment. Consistently, we observed increased endometrial gelatinolytic activity of MMP-2 compared to MMP-9 activity and thus a higher ratio of MMP-2/MMP-9 gelatinolytic activity in the endometrium of the cranial segment compared to the caudal one.

The important role of TIMPs as regulators of MMP-2 and MMP-9 activities has been clearly established in many developmental tissues [16,56]. TIMPs play a central role in limiting the extent of ECM degradation, and both TIMP-1 and TIMP-2 possess anti-invasive and anti-angiogenic properties during the tissue-remodelling process [57]. In our study, the endometrial localisation of TIMP-1 and TIMP-2 was similar to that observed in the human uterus [58] and in bovine females [59]. TIMP-1, an inhibitor of MMP-9 activity, and TIMP-2, the main inhibitor of MMP-2, were mainly increased in the luminal and glandular epithelium of the caudal segment. However, a high TIMP-1/TIMP-2 ratio was observed in the cranial endometrium, which seems to be due to the low TIMP-2 content. This, together with the high MMP-2 activity, could be related to the MMP-2/MMP-9 activity ratio observed by zymography. Therefore, we believe that the ECM remodelling of the cranial segment consists of an important degradation and rupture of the fibrillar components, mainly due to the action of MMP-2. However, further research is needed to determine whether changes in the expression of TIMPs are linearly related to their inhibitory activities. Post-transcriptional modification may alter TIMPs but it is not yet known whether the inhibitory activity in the non-pregnant endometrium is the same as for other proteins [54].

On the other hand, since TIMPs play central roles in ECM degradation during tissue-remodelling processes [60], and an appropriate MMPs/TIMPs balance is needed for successful implantation and placentation [54,61,62], an adequate relation between MMPs and TIMPs may also be required to regulate the MMP activities in the non-pregnant uterus of vizcacha. MMP-2/TIMP-2 expression ratio increases, and the ratio of MMP-9/TIMP-1 expression tends to decrease in the cranial segment, because of increased expression of TIMP-1. Consequently, we believe that in this endometrial segment the degree of degradation of collagen fibrillar components is higher than in the caudal one, and therefore the rate of remodelling of superficial and deep endometrial ECM is greater. Contrarily, the higher content of collagen and elastic fibres and probably a higher organisation/arrangement of collagen fibres in the connective tissue of the caudal segment of the endometrium, as mentioned above, suggest a lower rate of remodelling.

The importance of the balance and regulation of MMP-2 and MMP-9 expression and activity was also previously observed in mouse decidua at day 10 of gestation [23]. In our study, the relatively lower activity of MMP-9 compared to MMP-2 activity in the caudal segment, compared to the middle and cranial segments, could lead to partial degradation of MMP-2-denatured collagen type I and III fibres in the deep endometrium of the caudal segment, which is why they were observed to be quantitatively increased by Picrosirius. Consistent with this idea, previous biochemical studies show the presence of collagen types I and III in the isolated endometrium of non-pregnant mice [63], but a significant and rapid increase in collagen fibril thickening of diameter is required for decidualisation to occur during pregnancy, through the interaction and addition of collagen III to the main structural component collagen I [64,65]. In the non-decidualised area, on the other hand, close to the myometrium, the collagen fibrils remain thin throughout pregnancy [66].

Based on the above, we believe that the development of the endometrial ECM of the caudal segment in the non-pregnant uterus of the viscacha requires a finely regulated balance between the expression and activity of both MMPs to precisely control the synthesis and remodelling of the uterine ECM prior to pregnancy. In addition to their proteolytic effects on ECM proteins, MMPs may also affect uterine and vascular function and the mechanisms of smooth muscle contraction. As some authors have shown that MMP-2 and -9 cause relaxation of the precontracted rat uterus [67], regulation of the expression and activity of both MMPs may be crucial for an adequate physiological control of the non-pregnant endometrial uterus for a potential pregnant state. For the first time, these results could contribute and help to explain the previously observed greater embryo survival at the caudal segment of viscacha’s pregnant uterus [33,68].

Finally, we consider that our results, in combination with those previously obtained [5,6,7], show that the uterine horns of non-pregnant viscachas exhibit morphological variations that could lead to differential growth and development among IS, potentially explaining embryonic mortality. We rule out that the findings in the ECM are due to variable hormonal inductions that could result from differential vascularisation along the uterine horns. This conclusion is based on the fact that all the analysed females were in anestrus and that, during this period, non-pregnant females have the lowest baseline progesterone levels compared to pregnant females [69].

These findings in the ECM, as well as those identified previously, will allow for further exploration of this embryonic death in the species and contribute to a comprehensive understanding of it.

## 5. Conclusions

The results indicate that endometrial remodelling in the uterine horns of non-pregnant vizcachas varies in the tissues of the uterine endometrium in a longitudinal craniocaudal direction and suggest that such variation may be related to the embryonic loss at the cranial and middle IS while embryo survival is maintained at the caudal sites. These studies provide a first approximation to the knowledge of factors and mechanisms of the intrauterine environment related to changes in the remodelling of the endometrial ECM that determine early, sectorised, massive, and spontaneous embryonic death in the viscacha. Future studies conducted with pregnant female viscachas, at different gestational stages, will allow us to verify these hypotheses about the role of the ECM in embryonic death in this species.

## Figures and Tables

**Table 1:** Staining, observation, and interpretation of the fibres studied.

Fibres	Staining	Observation	Interpretation
Collagen fibres	Van Gieson	BF	Red-pink
	Picrosirius	BF	Red-pink
	Picrosirius	PL	Red-yellow (type I)
			Green (type III)
Elastic fibres	Verhöeff-Van Gieson	BF	Terracota

CT, connective tissue; BF, bright fiel microscopy; PL, polarised light microscopy.

**Table 2:** Primary and secondary antibodies: dilutions and specifications.

Primary Antibodies			Secondary Antibodies		
Name	Dilution	Specifications	Name	Dilution	Specifications
MMP-2	1/50	Abcam, ab74277	LSBA kit	Ready to use	BioGenex, HK340. San Ramon, CA, USA
MMP-9	1/200	Abcam, ab74277			
TIMP-1	1/100	Santa Cruz Biotechnology, sc-6832R			
TIMP-2	1/400	Abcam, ab1828			
TIMP-3	1/100	Santa Cruz Biotechnology, sc-9906	Biotinylated goat anti-rabbit IgG	1/200	Vector, BP-9100-50. Burlingame, CA, USA
TIMP-4	1/50	Santa Cruz Biotechnology, sc-9374			

MMP, metalloproteinase; TIMP, tissue inhibitors of metalloproteinases (TIMPs).

## Data Availability

The original contributions presented in this study are included in the article. Further inquiries can be directed to the corresponding authors.

## Supplementary Materials

The following supporting information can be downloaded at: https://www.mdpi.com/article/10.3390/ani15040542/s1. Figure S1: Representative images of sections from the three uterine segments of non-pregnant *L. maximus*, stained with Picrosirius red and observed with bright-field microscopy to identify collagen fibres in the connective tissue of the endometrium; Figure S2: Negative controls of MMP-2 and MMP-9 in the superficial and deep endometrial zones of the cranial, middle, and caudal uterine segments of *L. maximus*; Figure S3: Negative controls of TIMP-1 and TIMP-2 in the superficial and deep endometrial zones of the cranial, middle, and caudal uterine segments of *L. maximus*; Figure S4: Immunostaining of TIMP-3 and TIMP-4 in the superficial and deep endometrial zones of the cranial, middle, and caudal uterine segments of *L. maximus*; Figure S5: Negative controls of TIMP-3 and TIMP-4 in the superficial and deep endometrial zones of the cranial, middle, and caudal uterine segments of *L. maximus*.
