# Supplementary material for: Differential Remodelling of Endometrial Extracellular Matrix in the Non-Pregnant Uterus of Lagostomus maximus as a Potential Mechanism Underlying Embryonic Death
Source: Animals (Basel). 2025 Feb 13;15(4):542. doi: 10.3390/ani15040542 (PMC11851369; doi:10.3390/ani15040542)
Supplement: Supplementary file 1 [file animals-15-00542-s001.zip › Figure S1.pdf]

Figure S1

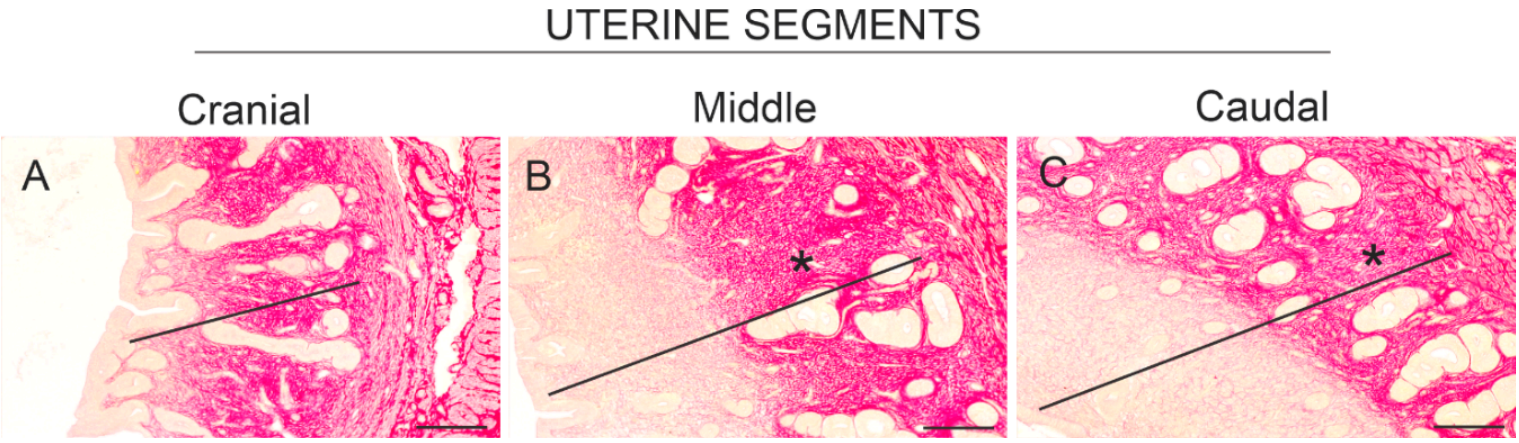

Figure S1. Representative images of sections from the three uterine segments of non-pregnant *L. maximus*, stained with Picrosirius red and observed with bright-field microscopy to identify collagen fibres in the connective tissue of the endometrium (solid line). A. Image showing collagen fibres homogeneously distributed throughout the thickness of the endometrium in the cranial uterine segment. B and C. Images showing collagen fibres predominantly distributed in the deep region (asterisk) of the endometrium in the middle (B) and caudal (C) segments. Scale bar: 200 μm.
