# Supplementary material for: Differential Remodelling of Endometrial Extracellular Matrix in the Non-Pregnant Uterus of Lagostomus maximus as a Potential Mechanism Underlying Embryonic Death
Source: Animals (Basel). 2025 Feb 13;15(4):542. doi: 10.3390/ani15040542 (PMC11851369; doi:10.3390/ani15040542)
Supplement: Supplementary file 1 [file animals-15-00542-s001.zip › Figure S3.pdf]

Figure S3

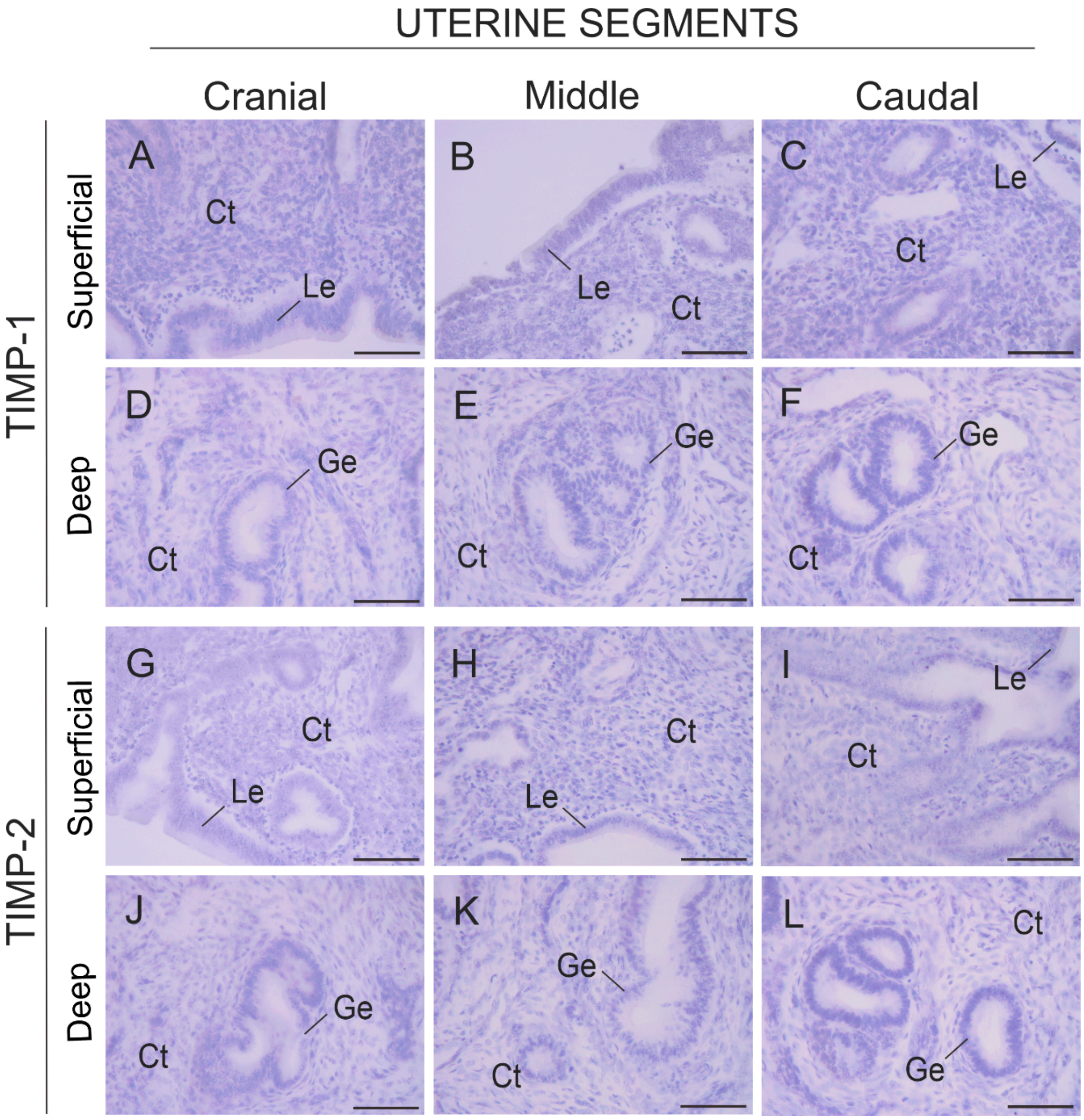

Figure S3. Negative controls of TIMP-1 and TIMP-2 in the superficial and deep endometrial zones of the cranial, middle, and caudal uterine segments of *L. maximus*. A-C. Immunostaining of TIMP-1 in the luminal epithelial tissue and connective tissue of the superficial endometrium from the cranial (A), middle (B), and caudal (C) uterine segments. D-F. Immunostaining of TIMP-1 in the glandular epithelial tissue and connective tissue of the deep endometrium in the cranial (D), middle (E), and caudal (F) uterine segments. G-I. Immunostaining of TIMP-2 in the luminal epithelial tissue and connective tissue of the superficial endometrium in the cranial (G), middle (H), and caudal (I) uterine segments. J-L. Immunostaining of TIMP-2 in the glandular epithelial tissue and connective tissue of the deep endometrium in the cranial (J), middle (K), and caudal (L) uterine segments. Scale bar: 200 μm. Abbreviations: Ct, connective tissue; Ge, glandular epithelium; Le, luminal epithelium.
