# Supplementary material for: Differential Remodelling of Endometrial Extracellular Matrix in the Non-Pregnant Uterus of Lagostomus maximus as a Potential Mechanism Underlying Embryonic Death
Source: Animals (Basel). 2025 Feb 13;15(4):542. doi: 10.3390/ani15040542 (PMC11851369; doi:10.3390/ani15040542)
Supplement: Supplementary file 1 [file animals-15-00542-s001.zip › Figure S4.pdf]

Figure S4

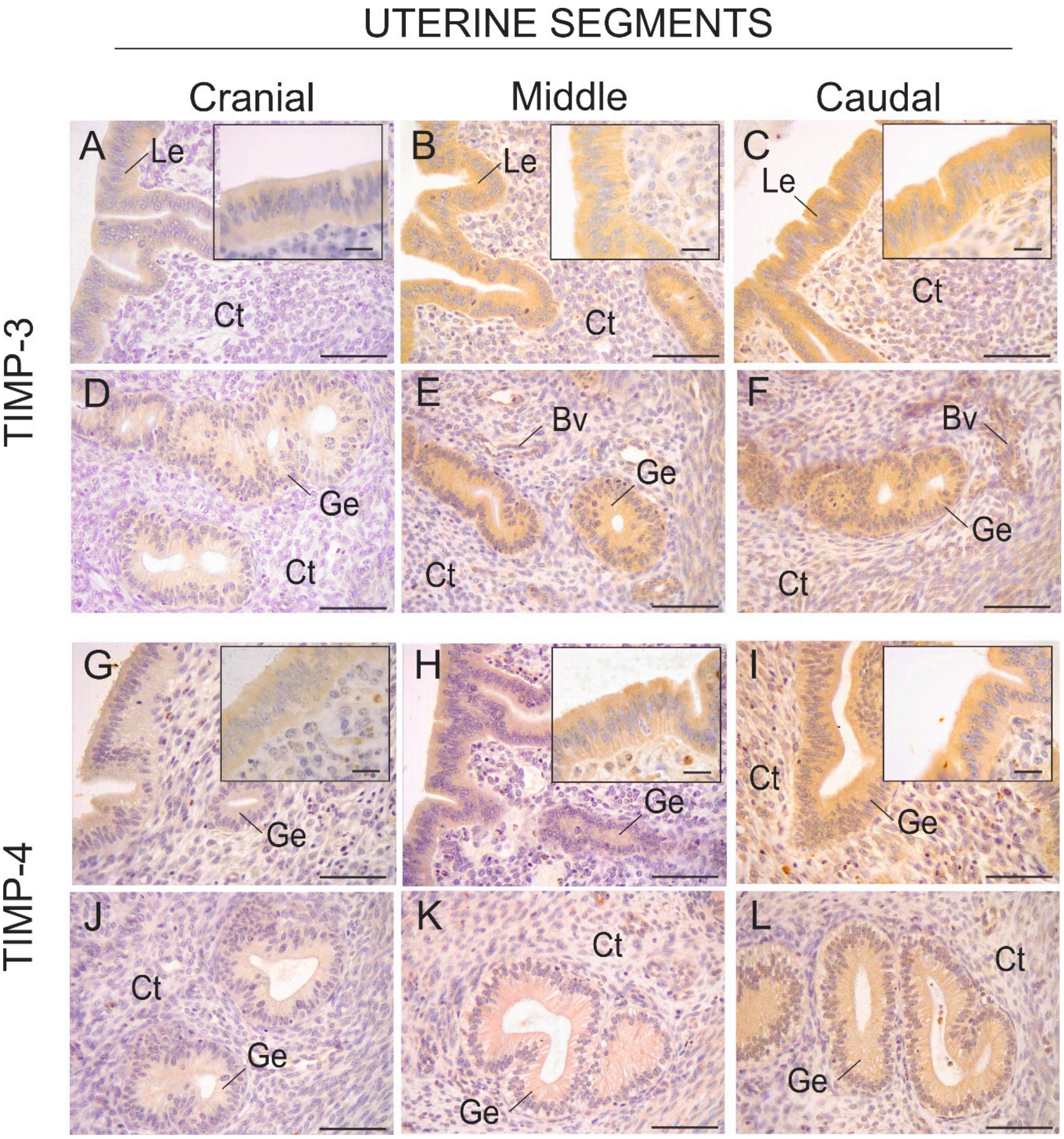

Figure S4. Immunostaining of TIMP-3 and TIMP-4 in the superficial and deep endometrial zones of the cranial, middle, and caudal uterine segments of *L. maximus*. A-C. Immunostaining of TIMP-3 in the luminal epithelial tissue and connective tissue in the superficial endometrium of the cranial (A), middle (B), and caudal (C) uterine segments. Inserts show high magnification of the epithelium. D-F. Immunostaining of TIMP-3 in the glandular epithelial tissue and connective tissue in the deep endometrium of the cranial (D), middle (E), and caudal (F) uterine segments. G-I. Immunostaining of TIMP-4 in the luminal epithelial tissue and connective tissue in the superficial endometrium of the cranial (G), middle (H), and caudal (I) uterine segments. Inserts show high magnification of the epithelium. J-L. Immunostaining of TIMP-4 in the glandular epithelial tissue and connective tissue in the deep endometrium of the cranial (J), middle (K), and caudal (L) uterine segments. Scale bar: 200 μm

(A-L), 20 μm (Inserts). Abbreviations: Bv, blood vessels; Ct, connective tissue; Ge, glandular epithelium; Le, luminal epithelium.
